# *Brachypodium distachyon* – A Useful Model in the Qualification of Mutagen-Induced Micronuclei Using Multicolor FISH

**DOI:** 10.1371/journal.pone.0170618

**Published:** 2017-01-24

**Authors:** Arita Kus, Jolanta Kwasniewska, Robert Hasterok

**Affiliations:** Department of Plant Anatomy and Cytology, Faculty of Biology and Environmental Protection, University of Silesia in Katowice, Katowice, Poland; Universite Laval, CANADA

## Abstract

*Brachypodium distachyon* (Brachypodium) is now intensively utilized as a model grass species in various biological studies. Its favorable cytological features create a unique foundation for a convenient system in mutagenesis, thereby potentially enabling the ‘hot spots’ and ‘cold spots’ of DNA damage in its genome to be analyzed. The aim of this study was to analyze the involvement of 5S rDNA, 25S rDNA, the Arabidopsis-type (TTTAGGG)n telomeric sequence and the Brachypodium-originated centromeric BAC clone CB33J12 in the micronuclei formation in Brachypodium root tip cells that were subjected to the chemical clastogenic agent maleic hydrazide (MH). To the best of our knowledge, this is the first use of a multicolor fluorescence *in situ* hybridization (mFISH) with four different DNA probes being used simultaneously to study plant mutagenesis. A quantitative analysis allowed ten types of micronuclei, which were characterized by the presence or absence of specific FISH signal(s), to be distinguished, thus enabling some specific rules governing the composition of the MH-induced micronuclei with the majority of them originating from the terminal regions of chromosomes, to be identified. The application of rDNA sequences as probes showed that 5S rDNA-bearing chromosomes are involved in micronuclei formation more frequently than the 25S rDNA-bearing chromosomes. These findings demonstrate the promising potential of Brachypodium to be a useful model organism to analyze the effects of various genotoxic agents on the plant nuclear genome stability, especially when the complex FISH-based and chromosome-specific approaches such as chromosome barcoding and chromosome painting will be applied in future studies.

## Introduction

Information regarding the distribution of DNA damage in plant chromosomes may be of great importance in understanding the biological impact of environmentally induced genotoxic effects. Furthermore, knowledge about the localization of chromosome rearrangements is crucial in plant mutagenesis, which is of practical value since most mutant varieties are developed using this approach. Cytomolecular identification and localization of chromosome breakage in plant chromosomes is possible using fluorescence *in situ* hybridization (FISH) [1, 2]. This technique provides the opportunity to detect even small aberrations in both dividing and non-dividing cells. Additionally, when FISH is used with the micronuclei (MN) test, it provides a better understanding of the micronuclei origin [3]. The effects of various mutagens have been analyzed in large-genome grass species, such as *Hordeum vulgare* (barley), in order to determine the origin of both physically [4] and chemically induced [1, 5] micronuclei. Similar studies have also been performed after maleic hydrazide (MH) treatment in a long-term *in vitro* root culture of *Crepis capillaris*, which is a weedy representative of Asteraceae [3]. For most of the plant species there is the dearth of FISH probes that can target individual chromosomes in a specific manner. Therefore, the distribution of chromosome aberrations in such species has as yet only been analyzed using such probes, as ribosomal DNA (rDNA) and *Arabidopsis thaliana* (Arabidopsis)-type (TTTAGGG)n telomeric sequences. Although these probes usually have no chromosome specificity, they are relatively widely applied because of their evolutionarily conserved nature and thus ‘universal’ character [1, 6, 7].

Multicolor FISH (mFISH) with the simultaneous application of more than two differently labeled DNA probes is widely used in mammalian cytogenetics, including mutagenesis and carcinogenicity analyses [8, 9] that dates back to the pioneering work of Nedorlof et al. [10], who simultaneously visualized three various sequences in human blood lymphocyte and tumor cells using different colors. Besides mammalian studies, mFISH has also found some, though rather limited, applications in plant cytogenetics [11–13]. However, to date it has not been used in mutagenesis studies involving plant material. The more widely used reprobing is based on at least two sequential FISH experiments applied to the same preparation usually using pairwise sets of probes that are removed after each experiment and replaced by another set [14]. This strategy has previously been successfully utilized to study the chromosomal aberrations and micronuclei formation in barley that had been subjected to chemical mutagens [5].

Numerous effective genotoxicity assays for the identification of DNA reactive compounds have been developed [15]. Due to the highly conserved structure of their genetic material, in theory it is possible to use a broad variety of plant species in genotoxicity tests [16]. However, *Brachypodium distachyon* (Brachypodium) is now a widely accepted model species for temperate cereals and forage grasses that has numerous favorable features and resources, such as a small, simple and fully sequenced nuclear genome, small plant stature and rapid life cycle. These are complemented by the availability of robust transformation protocols, tools for forward and reverse genetics screens and diverse germplasm collections, which have made this species a reference organism that is useful in studying various aspects of plant biology (for recent reviews see [17, 18]). The small number (x = 5) of relatively diverse chromosomes along with the low repeat content (21%) in its compact genome provides the unique opportunity to perform very precise cytomolecular analyses in Brachypodium [19]. It was demonstrated by Vogel and Hill [20] that Brachypodium is a promising plant for mutagenesis studies. Highly efficient *Agrobacterium*-mediated transformation systems have been developed for a range of Brachypodium genotypes [20, 21], including the reference diploid line Bd21 [22, 23]. Protocols for the mutagenesis of Brachypodium with sodium azide [24, 25], fast neutron radiation [26] and gamma radiation [27] have also been created. The positive response of Brachypodium to various mutagenic treatments, which is considered to indicate its suitable sensitivity, could also make this species useful in environmental monitoring. Although Brachypodium itself has no agricultural value and is not easily introgressed into other cereals, such an exploitation of this species would make it worthwhile to use it in various crop breeding programs with the potential to also be a model organism in plant mutagenesis.

In this study, we present the involvement of various chromosome fragments in micronuclei formation in Brachypodium for the first time and attempt to link them with the rules of the distribution of MH-induced changes in its nuclear genome. MH, which is chemically defined as 1,2-dihydro-3,6-pyridazinedione, is a clastogenic and mutagenic agent that may cause spindle fiber defects [28]. By examining the distribution of four different FISH probe signals (5S rDNA, 25S rDNA, Arabidopsis-type (TTTAGGG)n telomeric sequence and Brachypodium-originated centromeric BAC (Bacterial Artificial Chromosome) clone CB33J12) simultaneously, we quantitatively analyzed the micronuclei origin and formation.

## Materials and Methods

### Plant material and MH treatment

Seeds of *B*. *distachyon* (Brachypodium; 2n = 10) reference genotype Bd21 were presoaked in distilled water for 6 hours and then treated with an aqueous solution of maleic hydrazide (MH; Sigma-Aldrich) for 3 hours at two different concentrations of MH– 3 mM and 4 mM, respectively. Next, the seeds were washed 3×5 min in distilled water and then germinated in Petri dishes lined with moist filter paper for 3 days at room temperature in the dark. Whole seedlings were immersed in ice-cold water for 24 hours, fixed in 3:1 (v/v) methanol:glacial acetic acid and stored at -20°C until use. The experiment with MH treatment was repeated three times.

### Cytogenetic preparations

Cytogenetic preparations containing both mitotic chromosomes and interphase nuclei were made using a previously described procedure [29]. Briefly, the roots were cut off from the seedlings and washed in a 10 mM citric acid-sodium citrate buffer (pH 4.8), then digested enzymatically for 1.5 hours in a mixture containing 6% (v/v) pectinase (Sigma-Aldrich), 1% (w/v) cellulase ‘Onozuka R-10’ (Serva) and 1% (w/v) cellulase (Sigma-Aldrich). Root tips were squashed in a drop of 45% acetic acid. After freezing on dry ice, the cover slips were removed and the preparations were air dried and stored at 4°C until use.

### Probes and labeling

The following probes were used in this study:

1. Clone pTa794 containing 5S rDNA isolated from *Triticum aestivum* [30] was labeled with biotin-16-dUTP (Roche Diagnostics) using PCR and the procedure described by Hasterok et al. [11].
2. Clone HT100.3 containing 30 copies of Arabidopsis-type (TTTAGGG)_n_ telomeric repeats [31] was labeled like the previous probe using PCR but with digoxygenin-11-dUTP (Roche Diagnostics).
3. Centromeric BAC clone CB33J12 was isolated from *Escherichia coli* using a standard alkaline lysis method as described by Farrar and Donnison [32] and labeled with tetramethylrhodamine-5-dUTP (Roche Diagnostics) using a nick translation mix (Roche Diagnostics) according to Jenkins and Hasterok [29].
4. The clone containing a 2.3 kb *Cla*I fragment of the 25S rRNA gene of Arabidopsis [33] was labeled with digoxigenin-11-dUTP (Roche Diagnostics) or tetramethyl-rhodamine-5-dUTP (Roche Diagnostics) in two separate labeling reactions using nick translation. After labeling, the probes were mixed in equal ratios and precipitated. These probes were used to detect the 35S rDNA loci.

### Fluorescence *in situ* hybridization, image acquisition and processing

The FISH procedure was adopted from Jenkins and Hasterok [29] with minor modifications. In brief, the slides were pre-treated with RNase, washed several times in a 2×saline sodium citrate (SSC) buffer, dehydrated in an ethanol series and air-dried. The hybridization mixture, which consisted of 50% of deionized formamide, 10% dextran sulphate, 2×SSC, 0.5% SDS and 2.5–3.0 ng/μl of labeled DNA, was predenaturated at 75°C for 10 min, applied to the slides and denatured together with the cytological material at 70°C for 4.5 min and allowed to hybridize overnight at 37°C in a humid chamber. Post-hybridization washes were performed in 20% deionized formamide in 2×SSC for 10 min at 37°C (the equivalent to a 59% stringency; the degree of homology between a probe and target DNA). Deoxygenated probes were detected using FITC-conjugated anti-digoxigenin antibodies (Roche Diagnostics), while the biotinylated probe was detected using Alexa Fluor 647-conjugated anti-biotin antibodies (Jackson Immuno Research). After final washes in Tween20/4×SSC followed by ethanol dehydration, the air-dried preparations were mounted and counterstained in Vectashield (Vector Laboratories) containing 2.5 μg/ml DAPI (Serva).

The preparations were examined using a Zeiss AxioImager.Z.2 wide-field fluorescence microscope. All of the images were acquired with an AxioCam MRm monochromatic camera (Zeiss) and then processed using Photoshop CS3 (Adobe). The frequencies of micronuclei with specific signals and without signals were calculated. For each experimental group, a total of 150 nuclei with micronuclei were evaluated. Three slides, each one made from one meristem, were analyzed. In order to estimate the total frequency of DAPI-stained micronuclei, at least 2,000 cells were analyzed on the same slides for each experimental group before the FISH experiments.

## Results and Discussion

### Brachypodium as a model in mutagenesis

**Fig 1:**
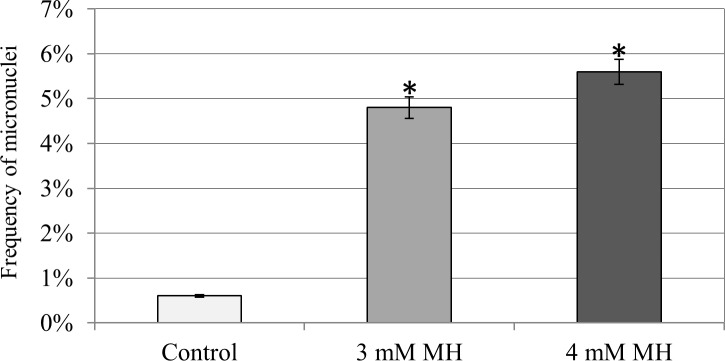
Frequencies of micronuclei in Brachypodium root tip meristematic cells after two concentrations of MH treatment. The bars show the standard deviation and the asterisks indicate the groups that were significantly different from the control at P<0.05 using the Student’s t-test.

The treatment of Brachypodium seeds with 3 mM or 4 mM MH caused a prominent clastogenic effect, which was manifested by the presence of micronuclei in the cells at interphase. Analysis of the frequencies of the cells with micronuclei was done using DAPI staining. The frequency of the cells with micronuclei depended on the mutagen concentration and varied from 4.8% for 3 mM MH to 5.6% for 4 mM MH (Fig 1). To the best of our knowledge, this is the first study on the susceptibility of Brachypodium to mutagenic treatment linked with FISH-based qualitative and quantitative analyses of the effects of such a treatment on the nuclear genome stability visualized at the cytomolecular level. The mutagenic effect of MH has been well known for a longtime [34]. Although there are no published data available on the chemically induced clastogenic effect for Brachypodium, the mutagenic effect of MH has previously been demonstrated for a wide of range other plant species such as *Crepis capillaris* [3], *Allium cepa* [28, 35, 36], *Vicia faba* [37] and *Tradescantia* [38]. In contrast, according to Gichner et al. [39], MH is not mutagenic in Arabidopsis cells. In barley, MH at a concentration of 3 mM induced the micronuclei at almost a two-fold higher frequency (8.8%) [5] than in Brachypodium (4.8%). This difference may be connected with the fact that barley has one of the largest cereal genomes (~5.1 Gb) [40], which is approximately 16× larger than the nuclear genome of Brachypodium (~311 Mb) [41]. Such a diverse response to the mutagen is consistent with the ABCW hypothesis, which states that the mutation rate per locus is proportional to the genome size of a species [42]. Therefore, due to its small genome size and high sensitivity, Brachypodium can serve as a potentially useful model system in mutagenesis studies.

### Chromosomal distribution of rDNA, centromeric and telomeric probes

**Fig 2:**
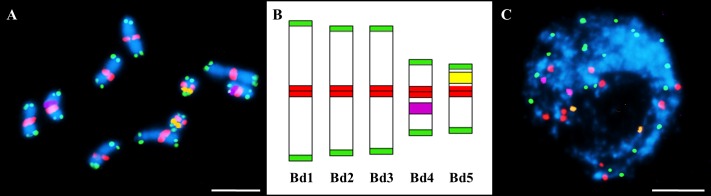
Multicolor FISH with 5S rDNA (purple), 25S rDNA (yellow), centromeric (green) and telomeric (red) probes on the untreated (control) material from Brachypodium root tips. **(A)** Mitotic metaphase chromosomes (2n = 10) with no aberrations visible. **(B)** Idiogram (x = 5) of Brachypodium chromosomes. **(C)** Exemplary interphase nucleus with no micronuclei. Bars: 5 μm.

Once the clastogenic effect was quantified, multicolor FISH with 5S rDNA (purple), 25S rDNA (yellow), telomeric (green) and centromeric (red) probes applied simultaneously was used to determine the involvement of specific chromosomes or their fragments in the formation of the MH-induced micronuclei. The use of three different labels and the simultaneous labeling of the 25S rDNA probe with two different labels enabled the unambiguous, four-color discrimination of all of the probes (Fig 2) and their visualization on mitotic metaphase chromosomes and in interphase nuclei. Two out of five pairs of Brachypodium chromosomes (Bd1-Bd5) are characterized by the presence of rRNA genes; the acrocentric chromosome Bd4 is known to carry the prominent 5S rDNA locus in the proximal part of its long arm while acrocentric and the smallest in the complement Bd5 has the 35S rDNA locus in the distal region and secondary constriction of its short arm [43]. The telomeric probe hybridized to the physical ends of all of the chromosomes and no intercalary signals were observed along any of the chromosomes in the complement, while the centromeric BAC specifically targeted the primary constrictions in all of the chromosomes (Fig 2A and 2B). The exemplary interphase nucleus from the untreated Brachypodium root tip showed two signals of 5S rDNA and 25S rDNA, ten centromeric and twenty telomeric signals (Fig 2C). The physical localization of the 5S rDNA and 35S rDNA sequences in Brachypodium provides useful chromosome landmarks for two of its five chromosome pairs, thus enabling the unambiguous discrimination of the Bd4 and Bd5 chromosomes. In contrast, four of seven chromosome pairs in barley are characterized by the localization of 5S rDNA while two other pairs are the NOR-bearing chromosomes [5]. The higher number of rRNA gene loci in the barley chromosome complement may hamper a precise determination of which chromosome or chromosome fragment(s) are involved in micronuclei formation, which is not the case for Brachypodium, where both rDNA markers are strictly chromosome-specific, and therefore allow the contribution of the Bd4 and Bd5 chromosomes in micronuclei formation to be determined.

Due to the low number of relatively large and highly diverse chromosomes and the favorable chromosomal distribution of rRNA gene loci, *C*. *capillaris* was used in the past for the assay of chromosome aberrations after MH treatment [3]. Despite its inborn advantages, *C*. *capillaris* suffers from a relatively poorly developed cytomolecular research infrastructure compared to Brachypodium, which significantly limits the usefulness of this species in any complex mutagenesis studies and reinforces the need to develop a more useful plant model, preferably one that is phylogenetically related to the large-genome temperate zone cereals and that can be extensively utilized in a plethora of other biological studies. There is no doubt that at present Brachypodium fulfills all of these requirements [17, 18].

### The distal parts of the chromosomes in Brachypodium are the most fragile regions after MH treatment

**Fig 3:**
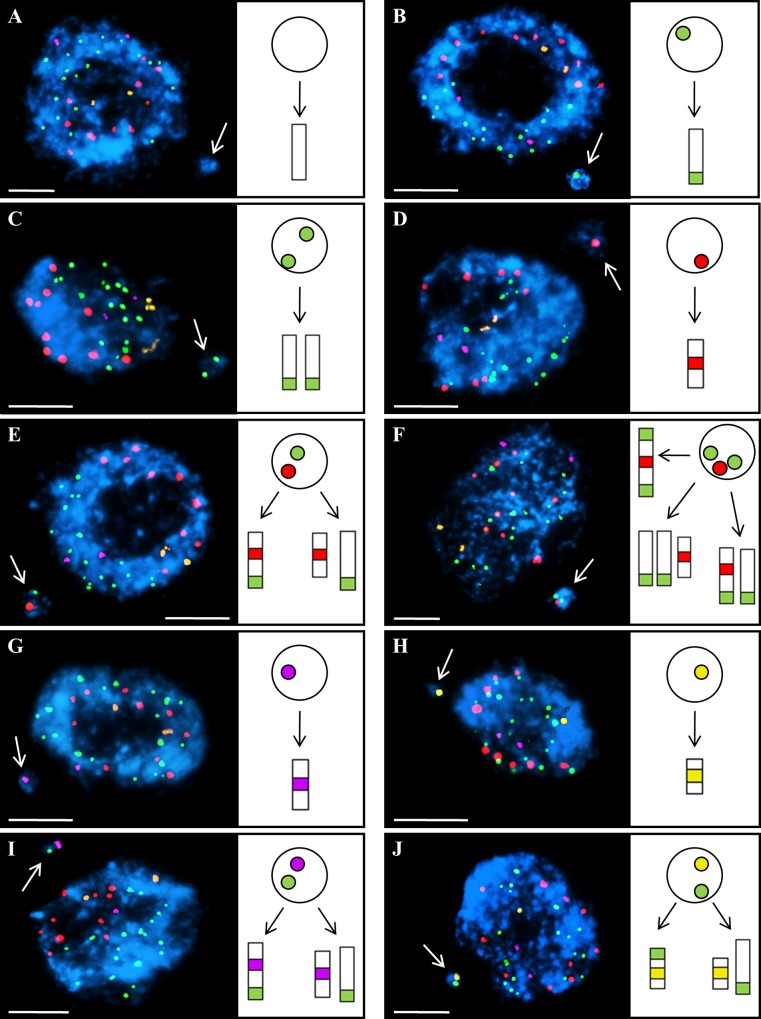
Interphase nuclei with micronuclei from Brachypodium root tips after MH treatment subjected to FISH with the same probe set as in Fig 2. Ten different classes of micronuclei are distinguished: **(A)** without any FISH signals, **(B)** with one telomeric signal, **(C)** with two telomeric signals, **(D)** with one centromeric signal, **(E)** with one centromeric and one telomeric signal, **(F)** with one centromeric and two telomeric signals, **(G)** with one 5S rDNA signal, **(H)** with one 25S rDNA signal, **(I)** with one 5S rDNA and one telomeric signal, **(J)** with one 25S rDNA and one telomeric signal. White arrows indicate the micronuclei. The diagrams next to the photomicrographs show the putative origins of the micronuclei. Bars: 5 μm.

In the material that was subjected to the MH treatment, cells with micronuclei of a variety signal compositions were observed (Fig 3). Based on this diversity, we distinguished ten categories of micronuclei that were characterized by the presence or absence of specific FISH signal(s). The application of 5S rDNA and 25S rDNA FISH probes enabled the involvement of the rDNA-bearing regions of Brachypodium chromosomes Bd4 and Bd5 in the micronuclei formation to be analyzed. The use of centromeric probes and telomeric probes allowed a determination of whether the entire chromosomes or only their fragments constitute the micronuclei. Since the micronucleus in Fig 3A does not contain any signals, it implies the involvement of an interstitial chromosome fragment of unknown origin in its formation.

The presence of one telomeric signal in a micronucleus (Fig 3B) indicates its origin from an acentric chromosome fragment. Micronuclei with two telomeric signals were also observed (Fig 3C), which suggests that two acentric fragments of an unknown origin were involved in their formation. By contrast, the micronucleus with one centromeric signal probably originated from the interstitial chromosome region (Fig 3D). The micronucleus presented in Fig 3E is characterized by the presence of one telomeric and one centromeric signal. There are two possibilities of its origin, either from one chromosome arm or from two unrelated fragments, one of which contained a centromere. The micronucleus in Fig 3F is composed of three signals–two of the telomeric and one of the centromeric probes. Such a composition implies several possible origins of its formation, i.e. from one complete, laggard Bd1, Bd2 or Bd3 chromosomes, from two chromosomes fragments or from three fragments–one centric and two acentric. The 5S rDNA-positive micronucleus in Fig 3G is an example of the involvement of the interstitial fragment of chromosome Bd4. By contrast, the micronucleus in Fig 3H originated from an interstitial fragment of chromosome Bd5 and involved the 35S rDNA locus. The micronucleus with a 5S rDNA signal and a telomeric signal in Fig 3I probably originated from an acentric fragment of chromosome Bd4. It is also possible that the interstitial fragment of chromosome Bd4 and the terminal fragment of another chromosome are involved in this micronucleus formation. Finally, we also observed a micronucleus that was characterized by the presence of 25S rDNA and telomeric signals (Fig 3J). This implies the involvement of an acentric or interstitial fragment of NOR-bearing chromosomes (Bd5) in its formation.

**Fig 4:**
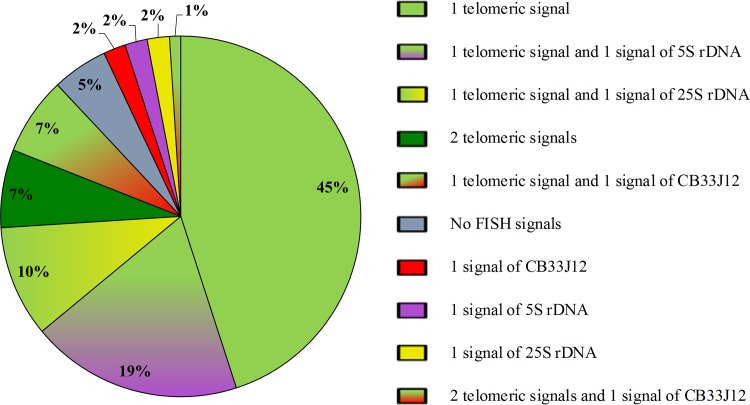
Frequencies of various micronuclei classes from Brachypodium root tips after MH treatment that carry specific DNA sequences. The data for two concentrations of MH have been combined.

In the present study, the application of mFISH enabled the frequencies of micronuclei with a particular FISH signal composition to be analyzed, thus allowing the preferred mechanism of their origin to be inferred (Fig 4). Because frequencies of micronuclei with the respective types and combinations of FISH signals did not depend on the concentration of MH, the data that was obtained for the two mutagen concentrations have been combined into one dataset. The quantitative analysis of all ten types of micronuclei that were found in Brachypodium root tip cells revealed that micronuclei with one telomeric signal were the most frequent and represented 45% of all of the micronuclei that were observed. Similar results were reported in barley cells by Jovtchev et al. [1], who showed that most N-nitroso-N-methylurea (MNU)-induced micronuclei were characterized by the presence of telomeric signals. In contrast, only 10% of MH-induced micronuclei with telomeric DNA sequences were reported in barley, though 46% of them had both telomeric and rDNA (5S or 25S) signals [5]. These results indicate that micronuclei in Brachypodium after MH treatment arise mostly from the terminal, acentric fragments of chromosomes, while in barley MH preferably leads to large acentric fragments including rDNA loci that are located in the interstitial parts of the chromosomes or near the centromere regions.

Micronuclei that contained one telomeric and one 5S rDNA signal were also relatively abundant (19%) in Brachypodium (Fig 4). In contrast, the eight remaining micronuclei categories were significantly scarcer since they collectively contributed to only 36% of all of those that were observed. The most frequent (10%) in this group were the micronuclei that displayed one telomeric and one 25S rDNA signal. These observations indicate that despite their similar size, Bd4 chromosomes are involved in the MH-induced micronuclei formation more often than Bd5 chromosomes. Similar results were observed in gamma ray-induced micronuclei in barley [4]. Moreover, an additional locus of 35S rDNA was present after MH treatment in barley cells, which suggests that either a duplication or double strand breaks (DBS) occurred within the 35S rDNA locus. Though the NOR regions were found to exhibit increased fragility both in some plants [44, 45] and in humans [46], we did not observe this in Brachypodium cells. A different sensitivity of 5S rDNA and 25S rDNA sequences was also reported in *C*. *capillaris* after MH treatment, where the application of comet-FISH demonstrated that 5S rDNA was present in the comet tail more frequently than 25S rDNA [47]. These authors postulated that an association with the nucleolus may make the 25S rDNA less prone to migration into the comet tail. In the present study, micronuclei with only one signal, either of the 5S rDNA or 25S rDNA probe, were observed with the same frequency of 2%, thus suggesting that interstitial breaks within the Bd4 and Bd5 chromosomes occurred relatively rarely.

There are some analyses that are based on calculating the centromere signal-positive and centromere signal-negative micronuclei that have been performed for barley [1, 5] and *C*. *capillaris* [3]. In addition to mutagenesis, this strategy has been also applied by dos Reis et al. in their study of tissue-specific instability in synthetic interspecific *Pennisetum* hybrids [48]. In the case of Brachypodium, 7% of the micronuclei with one telomeric and one centromeric signal were observed. Such a result may indicate an aneugenic action of the applied mutagen and would be in agreement with the results of some earlier studies [5]. The class of Brachypodium micronuclei that contained nothing but one centromeric signal was detected at a low frequency (2%), which was about 22 times lower than the frequency of micronuclei possessing one telomeric signal (45%; Fig 4). Such results indicate that the distal parts of the chromosomes in Brachypodium are the most fragile regions after MH treatment, even considering twice higher number of telomeric signals (20) than centromeric signals (10) in the Brachypodium control nuclei. Our data correspond to that reported by Jovtchev et al. [1], who showed that only 1% of MNU-induced barley micronuclei revealed centromere DNA signals. Interestingly, the frequency of such micronuclei in barley that was subjected to MH treatment was considerably higher (6%) [5], whereas they were not observed at all in barley cells after gamma radiation [4]. Such discrepancies in the frequencies may indicate that only MH and MNU cause interstitial chromosome breaks. Surprisingly, micronuclei with two telomeric signals were observed at a relatively high frequency in Brachypodium (7%), while this class of micronuclei was not present at all in barley after MH treatment [5], though it was reported in *C*. *capillaris* MH-induced micronuclei [3]. The least observed micronuclei were those that contained two telomeric signals and one centromeric signal, since they contributed to only 1% of the total number of micronuclei. The present results suggest that telomeric sequences are involved in the micronuclei formation more often than the centromeric ones, which is not surprising considering the fact that the formation of a micronucleus that contains one telomeric signal requires only one DSB, whereas two such breaks are needed to form a micronucleus with only a centromeric signal.

Interestingly, no micronuclei that contained both kinds of rDNA or centromeric and rDNA signals were identified. In contrast, MH-induced micronuclei with two 5S rDNA and two 25S rDNA specific signals were observed in barley cells at a low frequency (4%) [5].

### Conclusions

The use of maleic hydrazide, a potent chemical mutagen, and detection of its effect on Brachypodium nuclear genome stability using multicolor FISH with four simple probes demonstrated the usefulness of this model grass in plant mutagenesis. The cytogenetic findings presented here are essentially consistent with the findings that have been demonstrated for other species, both monocot [5] and dicot [3], and confirmed the following facts: (i) the distribution of chromosome aberrations is not random, (ii) the origin of micronuclei is often from the acentric fragments, (iii) the distal parts of chromosomes are more frequently involved in the micronuclei formation than the proximal ones. Although the mFISH approach that enabled the simultaneous application of four different probes is by itself quite novel in plant mutagenesis, it can be anticipated that the real potential of Brachypodium as a model in such studies using single-locus chromosomes specific BAC clones [49] and/or their sequence-ordered painting pools [50] will be developed in the near future. These BAC-FISH-based ‘chromosome barcoding’ and ‘chromosome painting’ approaches have proven to be effective in revealing the structure and evolution of Brachypodium karyotypes [51–53] and interphase nucleus organization [54, 55]. They are also very likely to provide a plethora of useful information about the exact mechanisms that govern micronuclei formation in plants after mutagenic treatment.
